# Driving into the Unknown: Investigating and Addressing Security Breaches in Vehicle Infotainment Systems

**DOI:** 10.3390/s26010077

**Published:** 2025-12-22

**Authors:** Minrui Yan, George Crane, Dean Suillivan, Haoqi Shan

**Affiliations:** 1 School of Science, Computing and Emerging Technologies, Swinburne University of Technology, Hawthorn Campus, Melbourne, VIC 3122, Australia; 2 Electrical and Computer Engineering, The University of New Hampshire, Durham, NH 03824, USA; george.crane@unh.edu (G.C.);; 3 Stellar Cyber, San Jose, CA 95131, USA; hshan@stellarcyber.ai

**Keywords:** vehicle security, automotive security, network security, vehicle infotainment systems, internet of vehicles (IoV), fuzzing, symbolic execution

## Abstract

The rise of connected and automated vehicles has transformed in-vehicle infotainment (IVI) systems into critical gateways linking user interfaces, vehicular networks, and cloud-based fleet services. A concerning architectural reality is that hardcoded credentials like access point names (APNs) in IVI firmware create a cross-layer attack surface where local exposure can escalate into entire vehicle fleets being remotely compromised. To address this risk, we propose a cross-layer security framework that integrates firmware extraction, symbolic execution, and targeted fuzzing to reconstruct authentic IVI-to-backend interactions and uncover high-impact web vulnerabilities such as server-side request forgery (SSRF) and broken access control. Applied across seven diverse automotive systems, including major original equipment manufacturers (OEMs) (Mercedes-Benz, Tesla, SAIC, FAW-VW, Denza), Tier-1 supplier Bosch, and advanced driver assistance systems (ADAS) vendor Minieye, our approach exposes systemic anti-patterns and demonstrates a fully realized exploit that enables remote control of approximately six million Mercedes-Benz vehicles. All 23 discovered vulnerabilities, including seven CVEs, were patched within one month. In closed automotive ecosystems, we argue that the true measure of efficacy lies not in maximizing code coverage but in discovering actionable, fleet-wide attack paths, which is precisely what our approach delivers.

## 1. Introduction

The rapid evolution of connected and automated vehicles (CAVs) is transforming transportation into a cooperative, data-driven ecosystem. Modern vehicles no longer operate in isolation [1]; instead, they participate in coordinated maneuvers, shared perception, and infrastructure–vehicle collaboration through vehicle-to-everything (V2X) communication [2]. Recent advances, such as multi-agent system (MAS)-based hierarchical architectures for cooperative control, demonstrate how deeply interconnected today’s automotive systems have become. In this landscape, the In-Vehicle Infotainment (IVI) system has evolved from a standalone entertainment module into a critical gateway linking user interfaces, vehicular networks, and cloud-based cooperative services.

As a result, IVI systems now reside at the intersection of automotive and information technology, playing a pivotal role in enhancing user experience while simultaneously expanding the attack surface. These systems offer a blend of entertainment and information services, such as navigation, audio/video playback, climate control, and hands-free calling, delivered through interactive touchscreens, physical buttons, and voice commands. According to recent reports, the global automotive infotainment market was valued at roughly $31 billion and used by 82% of passenger vehicles in 2022 [3], underscoring their ubiquity and strategic importance.

The core interface of IVI systems often encompasses touchscreens, physical buttons, and voice commands, powered by dedicated computers running on specialized software tailored for automotive use. The orchestration between hardware and software facilitates many services, including processing user inputs and rendering the desired outputs through the vehicle’s displays. It is unlikely that all drivers are familiar with automotive communication and convenience technologies, let alone the ability to debug and/or repair commonly faced issues when they occur. Hence, owners rely upon the built-in safety and security of this infrastructure.

However, the intersection of connectivity, multimedia, and smart functionalities within IVI systems introduces a complex landscape of security challenges. The connectivity of these systems to external networks and devices makes them potential entry points for malicious actors. For instance, vulnerabilities within unencrypted firmware can expose the system to unauthorized access and manipulation [4]. Similarly, vulnerable vendor backend servers, remote control capabilities, and connection with in vehicle sensors, present an entirely different dimension of security risks that jeopardize not only the integrity of the IVI system, but also the safety and privacy of the vehicle occupants. Moreover, the integration of IVI systems significantly contributes to the broader Internet of Vehicles (IoV) ecosystem, which further extends the attack surface due to the increased interconnectivity between vehicles and external networks. Such a landscape necessitates rigorous security analysis to mitigate the risks associated with the exploitation of these vulnerabilities.

To address this gap, we propose a problem-driven, cross-layer security analysis framework specifically designed to answer a pressing question in modern IoV ecosystems: Can an attacker leverage firmware-extracted secrets to gain high-impact control over cloud-managed vehicles? Our methodology integrates three stages, (1) firmware extraction to recover hidden endpoints and credentials, (2) symbolic execution to reconstruct exact HTTP request templates issued by the IVI, and (3) targeted fuzzing using these high-fidelity templates to uncover critical web vulnerabilities like server-side request forgery (SSRF) and broken access control. Crucially, this is not a generic scanner, but a purpose-built workflow grounded in automotive-specific threat modeling.

We apply this framework across seven production systems from diverse stakeholders, including OEMs (Mercedes-Benz, Tesla, SAIC, FAW-VW, Denza), a Tier-1 supplier (Bosch), and an ADAS vendor (Minieye). Our analysis reveals systemic weaknesses such as hardcoded credentials, public exposure of internal services, and broken access point names (APN) isolation. Notably, within the Mercedes-Benz IoV ecosystem, we constructed an end-to-end exploit chain that escalates from local firmware extraction to remote control of approximately six million vehicles, verified on six physical cars. In total, we identified 23 vulnerabilities, including seven that were assigned CVE identifiers, all of which were confirmed and patched within one month.

Given the closed and heterogeneous nature of automotive environments, we argue that the efficacy of security analysis should be measured not by traditional metrics like code coverage, but by the discovery of actionable, end-to-end attack paths that expose real-world risks. Our work thus shifts the evaluation paradigm from isolated component testing to systemic IoV infrastructure assessment.

The contributions and findings of our work are summarized as follows:

1. We propose a cross-layer security analysis framework for IVI systems, motivated by the real-world architectural coupling between firmware-stored secrets and cloud backends. Our method systematically links firmware extraction, symbolic execution, and targeted fuzzing to uncover fleet-wide attack paths.
2. We demonstrate the framework’s effectiveness through a systematic evaluation across 7 automotive vendors, uncovering 23 vulnerabilities (7 CVEs) that reveal industry-wide anti-patterns, validating both the prevalence and severity of cross-layer threats.
3. We address in detail the vulnerabilities, impact, and potential mitigations affecting 7 automotive vendors. Meanwhile, we highlight the vulnerabilities affecting Mercedes-Benz in particular and summarize results for other vendors. Details of issues discovered for other vendors, including FAW-VW, Denza, SAIC, and Bosch, are presented in the Appendix A.
4. We present an end-to-end exploit against Mercedes-Benz that uses several new vulnerabilities with significant ramifications, e.g., enabling an attacker to gain back-end control of all connected vehicles in a web service, access to vehicle peripherals (locks, cameras, engine), and leaks privacy information on anyone registered on the IoV network.

## 2. Background and Related Works

In this section, we provide an overview of the Internet of Vehicles (IoV) ecosystem, focusing on the key components of an In-Vehicle Infotainment (IVI) system, the communication channels connecting these components, and the security risks associated with them. We will also delve into current IVI security research, comparing various studies to offer a well-rounded perspective. Additionally, we outline the threat model used in our investigation, preparing the ground for the analysis that follows.

### 2.1. IVI System and Security Architecture

**Figure 1:**
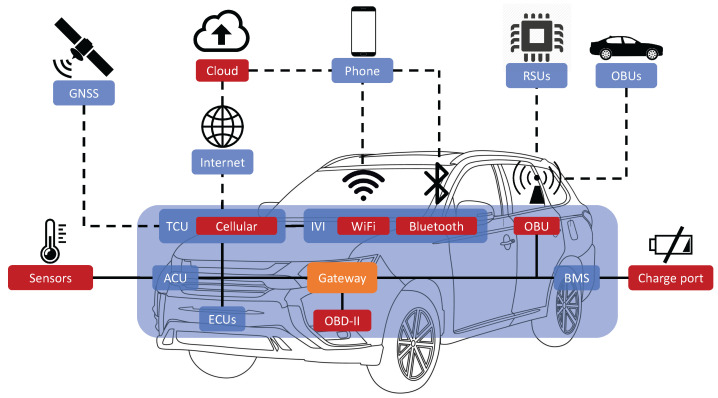
Common design of connected vehicles, which could be remote controlled by cloud servers or mobile devices.

The architecture of IVI systems is a sophisticated blend of hardware and software components meticulously orchestrated to provide a user-centric interface amidst a plethora of services, Figure 1. Central to this architecture is the processing unit, a dedicated computer that governs the operations of the IVI system, running specialized software tailored for automotive use. This unit acts as a hub, facilitating the interaction between the control interfaces—comprising physical buttons, touchscreens, and voice command systems—and the audio/video interfaces which deliver entertainment content. The connectivity modules embedded within the IVI system further extend its capabilities, enabling connections to external networks and devices via Wi-Fi, Bluetooth, and cellular connections. These modules not only enhance the functionality by allowing integration with external devices like smartphones or tablets but also enable communication with backend servers that manage data and services provided to the IVI system.

While the architecture of IVI systems contributes to enhanced user experience and functionality, it also unveils a spectrum of security threats. The external connectivity inherent in these systems can serve as potential gateways for unauthorized access and data breaches. Software vulnerabilities, if present, could be leveraged by malicious actors to gain control over the system or to inject malicious code. Insecure interfaces, both physical and wireless, if not adequately secured, present additional entry points for attackers. The challenge of ensuring robust authentication mechanisms further compounds the security concerns, allowing potential unauthorized access and manipulation if not addressed properly. In addition, the data privacy issues arising from the collection and transmission of personal or sensitive data underline the importance of implementing stringent security measures to safeguard the confidentiality, integrity, and availability of IVI systems and their data.

The security paradigm within IVI and IoV systems is tailored to mitigate risks both at the hardware level within vehicles and the web service layer provided by vendors. Central to this architecture is the harmonization between hardware safeguards and robust web security protocols. On the hardware front, aspects like secure boot and hardware security modules (HSMs) are crucial for ensuring the integrity and confidentiality of onboard data. Web security, on the other hand, is pivotal in securing the communication between IVI systems and vendor networks/servers. Beyond these, Access Point Name (APN) configurations, for instance, can provide a secure pathway into the vendor’s private network.

### 2.2. Access Point Name (APN)

APN is a critical configuration setting within cellular networks, acting as a gateway for devices, including those in IVI and IoV systems, to access the internet and other services provided by the mobile network operators. The APN configuration comprises essential information like the network identifier, operator’s IP address, and credentials, which are necessary for establishing the network connections. By defining the network path, an APN ensures that the devices can communicate externally while adhering to the required security and performance parameters.

In the context of IVI and IoV systems, a secure APN configuration is paramount to ensure securely managed communication between the vehicle and the external networks or servers. However, an incorrect or misconfigured APN can lead to unauthorized network access, as discovered in our research where extracted APN configurations provided a pathway into the vendor’s private network. This instance accentuates the importance of secure and robust APN configurations in preserving the security and integrity of the communications between IVI systems and external networks, forming a crucial facet of the overall security architecture.

### 2.3. Related Works

Security is important to the real world [5]. There have been several surveys [6,7,8] highlighting trends in (offensive) security research targeting automotive systems. A recent study [9] focuses exclusively on IVI systems security, in which they disclose their experiences from hosting an IVI hacking competition. The lessons learned reveal that not only are IVI systems vulnerable, but that vulnerable IVI system also adversely affect the user’s smart phone, vehicle, and even the user themselves. However, most prior related research attacking IVI systems have limited scope in that they only affect a single vehicle.

Miller and Valasek [10] showed the feasibility of remotely controlling an IVI system and other automotive features, e.g., HVAC, knobs, volume, etc., by jailbreaking the infotainment system and installing malicious firmware. Mazloom et al. [11] found that they could gain remote code execution on an infotainment OS via an attacker controlled smartphone. This was then used to trigger arbitrary commands on the internal network, including opening doors and starting the engine. In 2017, Jo et al. [12] were able to repackage maliciously modified firmware due to a misuse of certificates to gain remote control of the vehicle using SMSs. Research at the Tencent Keen Security Lab gained remote code execution and root privileges on the IVI system of a Tesla Model S and demonstrated vehicular control by injecting CAN messages. This work was extended to BMW, Mercedes-Benz and Lexus vehicles [13,14,15]. Remote code execution was demonstrated again on a Tesla Model 3 in the 2019 Pwn2Own competition [16] and used to control the vehicle. Subaru Starlink was shown to be vulnerable to malicious firmware modifications for attackers with physical access to the vehicle’s USB ports [17]. Constantin and Matteucci [18] exploit a vulnerability in the infotainment OS based on Android to upload a script that injects malicious commands onto the CAN bus. Mois and Alalfi [19] demonstrated that vulnerabilities in Android Automotive OS could be exploited to both leak information from and control an IVI system through a malicious application.

The running theme throughout these related works is undermining the security of the IVI system-via repackaged malicious firmware, arbitrary code execution, or injection of commands on a communication bus-to control the vulnerable vehicle. On the other hand, our work exploits similar physical and remote vulnerabilities to not only control the vehicle, but also, all vulnerable vehicles connected on the IoV network. We do so by gaining access to the backend server using a variety of methods, see Table 1, that provide a single point of entry for command-and-control to all vehicles on the vendor’s IoV. We estimate that, for a particular vendor, this would allow access and remote control to up to 6 million units.

### 2.4. Threat Model

We adopt a threat model aligned with established automotive security research [10], assuming an adversary with typical vehicle owner privileges. Specifically, the attacker:

- Has physical access to standard user-accessible interfaces, such as USB ports, SD card slots, or OBD-II connectors—commonly available to any car owner or technician;
- Possesses a valid user account for the vendor’s official mobile application or web portal (e.g., via legitimate ownership or credential theft);
- Does not require privileged hardware interfaces (e.g., JTAG, UART debug pins), firmware signing keys, or physical tampering beyond non-destructive disassembly.

This model reflects realistic attack scenarios where an adversary leverages local access to extract secrets (e.g., certificates, APN credentials) from a single vehicle, then uses these to launch remote, scalable attacks against the vendor’s backend infrastructure. Our goal is to evaluate whether local trust assumptions (e.g., “firmware is safe if encrypted”) can be weaponized to compromise the broader IoV ecosystem.

## 3. Methodology for Uncovering Cross-Layer IVI–IoV Vulnerabilities

Our investigation was motivated by a critical, yet underexplored, architectural trend in modern connected vehicles: the tight coupling between in-vehicle firmware and cloud-based backend services. We observed that manufacturers often embed sensitive network configurations—such as Access Point Name (APN) credentials—directly into IVI firmware to establish persistent, privileged connections to their private cloud infrastructure. These connections enable remote vehicle functions (e.g., unlocking doors, starting the engine), but also create a cross-layer attack surface: a vulnerability exposing firmware-stored secrets can directly lead to compromise of backend systems that control entire fleets.

Existing security analyses typically treat IVI firmware and web services in isolation—either auditing embedded code for local exploits or testing public APIs for logic flaws—thereby missing chained attacks that traverse both domains. To systematically investigate this risk, we designed a problem-driven methodology specifically tailored to uncover interdependencies between vehicle firmware and vendor backends.

Our approach proceeds in three tightly integrated stages, each addressing a key obstacle in exposing cross-layer vulnerabilities:

- Firmware Extraction recovers hidden API endpoints and embedded credentials (e.g., APNs) that are inaccessible via standard interfaces;
- Symbolic Execution reconstructs the exact structure of HTTP requests issued by the IVI system, enabling accurate modeling of backend interactions;
- Targeted Fuzzing leverages these high-fidelity request templates to probe backend services for critical web vulnerabilities (e.g., SSRF, authentication bypass).

**Figure 2:**
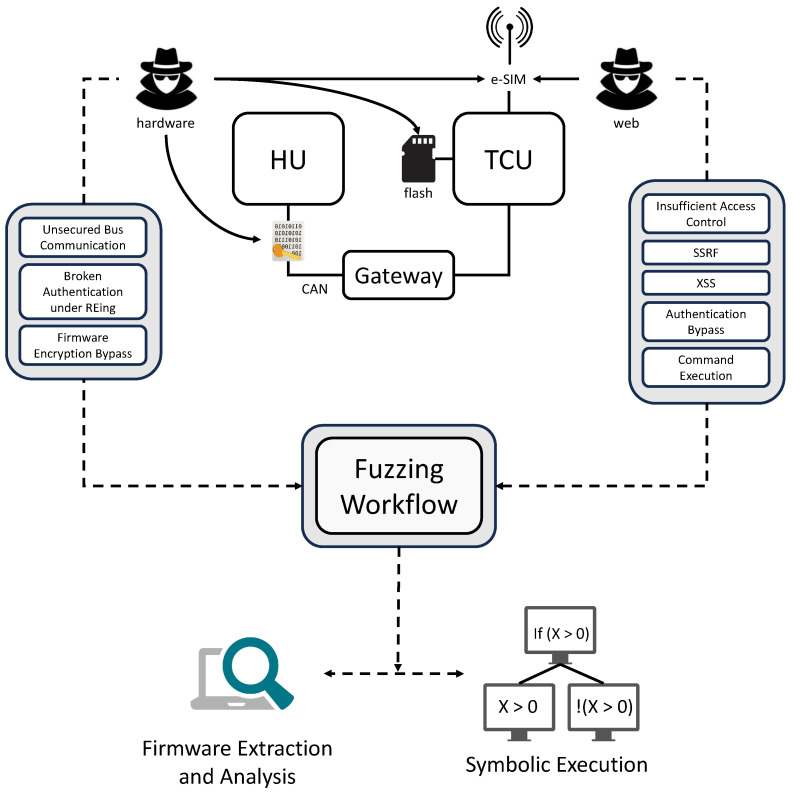
Methodology for uncovering cross-layer vulnerabilities in IVI–IoV systems. Starting from hardware access, we extract firmware to recover embedded backend endpoints and credentials (e.g., APNs). Symbolic execution then reconstructs valid HTTP request templates, which serve as seeds for precision fuzzing of vendor web services. This problem-driven workflow enabled the discovery of attack chains that escalate from single-vehicle firmware exposure to fleet-wide backend compromise.

Crucially, this pipeline is not a generic vulnerability scanner; it is purpose-built to answer a specific security question: Can an attacker leverage low-level firmware exposure to gain high-impact control over cloud-managed vehicle fleets? Given the increasing integration of vehicle firmware with centralized cloud infrastructure, this threat model represents a plausible and high-consequence attack vector that warrants systematic analysis. The overall workflow is illustrated in Figure 2.

### 3.1. Hardware Vulnerabilities

Hardware vulnerabilities pertain to the physical components and firmware that constitute the IVI system. They provide an avenue for adversaries to manipulate the inherent structure of the vehicle’s systems, thus compromising the security, safety, and functionality of the vehicle. The following sub-subsections delve into specific hardware vulnerabilities that are pertinent to vehicle security.

#### 3.1.1. Unsecured Vehicle Bus Communication

Unsecured vehicle bus communication arises when the data transmission over vehicle buses lacks proper security measures like encryption or authentication. This vulnerability could permit adversaries to eavesdrop or inject malicious messages, potentially leading to unauthorized control over critical vehicle functions, such as braking or steering, thereby posing serious safety threats.

#### 3.1.2. Broken Authentication Under Reverse Engineering

This occurs when authentication mechanisms can be bypassed or defeated through reverse engineering efforts. In the context of vehicle security, this vulnerability could expose sensitive system internals, thereby facilitating further attacks or unauthorized access to restricted vehicle functionalities, potentially compromising the security and safety of the vehicle and its occupants.

#### 3.1.3. Firmware Encryption Circumvention

Firmware encryption circumvention entails circumventing the encryption measures protecting the firmware, thereby gaining unauthorized access to the firmware’s code. This vulnerability could lead to exposure of sensitive vehicle information, unauthorized modification of the firmware, or exploitation of other vulnerabilities within the firmware, potentially impacting vehicle operation and user safety.

### 3.2. Web Vulnerabilities

These refer to the security flaws within web interfaces and services within which IVI systems interact. These vulnerabilities potentially expose the vehicle and its occupants to a myriad of risks ranging from privacy invasion to unauthorized control over vehicle functionality. The subsequent sections provide an insight into specific web vulnerabilities impacting vehicle security in our analysis.

#### 3.2.1. Insufficient Access Control

This occurs when users can perform actions or access data they aren’t supposed to due to inadequate or entirely absent access restrictions. Within a vehicle context, this vulnerability could lead to unauthorized access to sensitive vehicle data or control systems, potentially resulting in data breaches or unauthorized control over vehicle functionalities.

#### 3.2.2. Authentication Mechanism Bypass

Authentication mechanism bypass occurs when attackers can bypass the authentication mechanisms of a web application. This vulnerability could lead to unauthorized access to vehicle accounts or sensitive areas of the application, potentially resulting in data breaches, data alteration, or other malicious activities affecting the vehicle and its users.

#### 3.2.3. Server Side Request Forgery (SSRF)

SSRF is a vulnerability that allows an attacker to induce the server to make requests to other resources, potentially gaining unauthorized access to internal vehicle systems. This could lead to data exposure, internal network enumeration, or even remote code execution, thereby potentially compromising vehicle security and user safety.

#### 3.2.4. Cross-Site Scripting (XSS)

Cross-Site Scripting (XSS) occurs when an attacker can inject malicious scripts into content viewed by other users. In a vehicle security context, this vulnerability could lead to various attacks including stealing session cookies, spreading malware, or phishing, which may undermine the security and trustworthiness of vehicle web applications.

#### 3.2.5. Arbitrary Command Execution

Arbitrary command execution is a vulnerability where the attacker can execute commands on the host operating system through a vulnerable application. In vehicle systems, this can lead to complete system compromise, data theft, or denial-of-service, making it a critical security issue that necessitates rigorous validation and sanitation of input data, to ensure the safety and security of the vehicle and its occupants.

### 3.3. Web Fuzzing Framework

The increasing intricacy and inherent opacity of modern In-Vehicle Infotainment (IVI) systems present formidable challenges for comprehensive security assessment—a task of paramount importance given their deep integration with vehicle control and cloud services. Specifically, two core obstacles impede effective vulnerability discovery:

- Inadequate Coverage of Conventional Testing: Traditional approaches that interact with live IVI systems—such as triggering OTA updates or probing exposed APIs—often fail to exercise the full spectrum of internal logic. As a result, they overlook vulnerabilities that only manifest under specific, firmware-embedded conditions.
- Inadequate Coverage of Conventional Testing: Capturing genuine IVI-to-backend traffic in operational vehicles is frequently impractical due to encrypted channels, hardware-enforced isolation, and the absence of diagnostic interfaces during normal operation. Without these traces, reconstructing valid request formats for backend services becomes largely speculative.

These limitations highlight a fundamental gap: to effectively probe IVI-connected web services, one must first recover the exact request structures as generated by the firmware itself. Generic web fuzzers [20], while useful in other contexts, lack mechanisms to derive such high-fidelity seeds from closed automotive systems. Crucially, effective fuzzing of IVI web services demands precise knowledge of valid request structures, which are often heavily obfuscated, dynamically generated, and deeply embedded within the IVI firmware.

Therefore, our methodology necessarily begins with firmware-level analysis—not as a generic enhancement to fuzzing, but as an essential step to bridge the semantic gap between static code and dynamic cloud interactions. This enables targeted testing that bypasses the need for in-situ traffic capture while preserving protocol fidelity.

#### 3.3.1. Firmware Extraction and Analysis

The initial step in our fuzzing framework involves firmware extraction from a variety of manufacturers’ devices. Therefore, we categorize firmware into encrypted and unencrypted types. For encrypted firmware, we employ advanced techniques such as insecure bus communication and reverse engineering to facilitate extraction.

In-Vehicle Infotainment (IVI) systems primarily operate on general-purpose operating systems like QNX, Linux, or Android. We exploit the inherent file systems of these operating systems to analyze the extracted firmware’s partition tables to locate the binary offsets.

Post-extraction, we conduct a comprehensive scan of binary files to identify embedded strings that may contain potential target URLs, typically recognized by the ”http(s)://” substring. A custom-developed blacklist filters out irrelevant domains, while URLs containing manufacturer identifiers are logged for further analysis. A Euclidean distance-based ranking system is employed to prioritize the remaining strings against a predefined whitelist.

#### 3.3.2. Symbolic Execution for Web Request Parsing

Symbolic execution, underpinned by taint analysis, is utilized to dissect the parsed trajectory of web requests. The analysis centers around the send() function cluster, which serves as the conduit for an HTTP request dispatch. We delineate two core inquiries:

1. Identifying operations instrumental in manipulating the request.
2. Uncovering the final form of the request.

Addressing the first inquiry involves deploying a backward slicing technique from the send function to unveil all buffer manipulations. For the second inquiry, we encapsulate the buffer within a constraint for structured analysis. Consider the following code snippet in Listing 1. Backward slicing from send(buf) identifies all commands affecting buf. The snprintf function channels parameters to buf, and recursively, parameters v1, v2, v3 are identified from the preceding code.

**Listing 1.** Code snippet showing how GET/POST HTTP request are constructed and initiated in vehicle firmware.

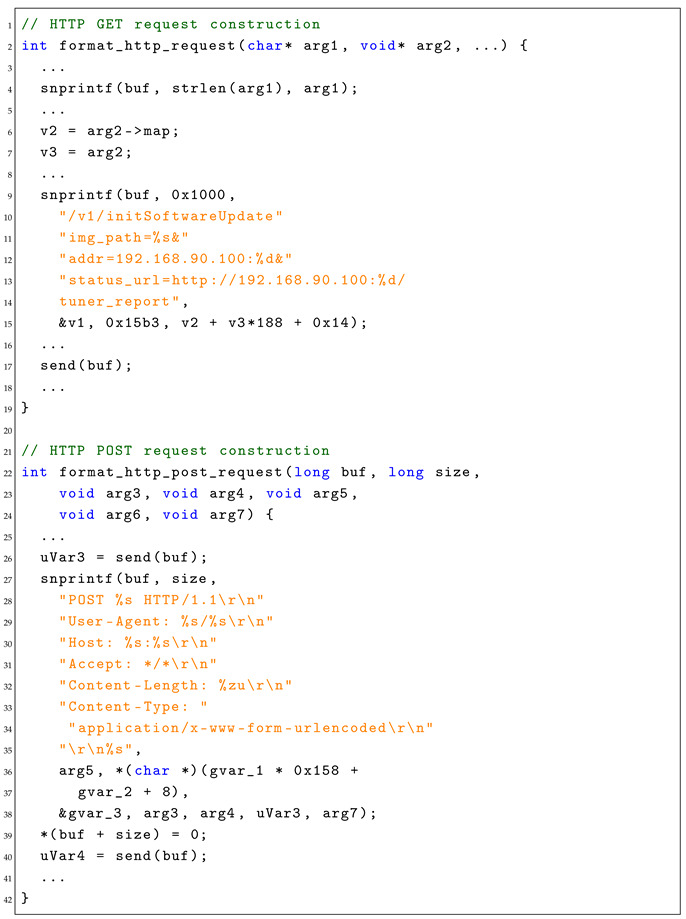



Constraint generation segments buf into discrete portions based on format patterns. These constraints stem from format string patterns, constant/fixed strings, and program logic aiding in the accurate reconstruction of web request structures. To precisely understand and represent the structure of the HTTP requests constructed by the firmware, it is crucial to dissect the buffer buf which carries the request. In this endeavor, we divide buf into different parts based on the format patterns inherent in the request construction logic. In our representative case, the divisions are as follows:

- original buf,
- /v1/initSoftwareUpdate?img_path=%s,
- &addr=192.168.90.100:%d,
- &status_url=http://192.168.90.100:%d,
- /tuner_report

The constraints that govern the formation of the request are deduced from the following:

1. The format string patterns, where “%s“ represents a string, and “%d“ represents an integer.
2. The constant/fixed strings present in the request.
3. The program logic; for instance, the first “%d” is a fixed number 0x15b3, while the second is derived from the expression $v_{2}+v_{3}\times 188+0x14$.

The dissection of buf into segments based on format patterns, coupled with the derived constraints, forms the bedrock for our subsequent request mutation and feedback processing phases, ensuring a structured and informed approach to fuzzing the firmware.

This symbolic reconstruction step is essential: in its absence, fuzzing would rely on uninformed input generation or partial network traces, both of which are unlikely to exercise critical endpoints—such as those governing vehicle telematics or remote actuation. Thus, symbolic execution serves as the critical bridge between static firmware analysis and dynamic web vulnerability discovery, enabling a truly cross-layer security assessment.

#### 3.3.3. Request Mutation and Feedback Processing

This phase encapsulates the mutation of requests and the analysis of feedback, underpinned by earlier identified parts and constraints. Mutations are confined within respective segments, adhering to format string patterns to maintain element integrity. Drawing inspiration from SNIPUZZ [4], we mutate constant, or fixed, strings and analyze coverage information from the response body to infer vulnerabilities. The feedback from these mutations, coupled with coverage information based on the observed responses, aids in iteratively refining the mutation strategies to uncover deeper vulnerabilities.

#### 3.3.4. Vulnerability Pattern Detection Through Fuzzing

With the knowledge gained from the preceding phases, our framework closely integrates vulnerability patterns into the fuzzing process, thereby amplifying its precision and effectiveness in unearthing web bugs.

A Holistic Strategy: Leveraging the established cases and mutation rules, our approach intertwines comprehensive exploration and targeted exploitation, ensuring not only that the surface is scanned but also that the deep paths are probed with specificity.

Targeted Vulnerability Discovery: Our framework weaves functionalities into mutation rules, thereby enhancing its capability to pinpoint typical vulnerability types. Below we delineate key modules embedded within our approach:

1. Crawler Module: Tasked with the discernment of diverse servers, directories, files, and unearthing directory traversal vulnerabilities, our crawler constructs URLs and paths to dynamically generate requests that probe potential weak spots. A case would be the incorporation of the string “?file://. /../etc/shadow“ appended to URLs, aimed at finding SSRF vulnerabilities. Furthermore, lateral movements are crafted utilizing information amassed during the firmware parsing phase, e.g., registered domain names, IP addresses, and web service architecture, thereby ensuring a multifaceted assault on potential vulnerability points.
2. XSS Module: Our framework also injects preset XSS strings into the request body, particularly when encapsulated within a text box, ensuring script injection points are tested.

Post-request dispatch, our framework analyzes the server responses, extracting and comparing pertinent data points such as status numbers, return sizes, and body information. This comparative analysis, grounded in our mutation and vulnerability patterns, allows us to discern discrepancies and anomalies that could potentially signal the presence of vulnerabilities. When a new functionality, such as an administrative login panel, is discovered, we generate a new set of test cases by analyzing and modifying a typical request generated by frontend functions.

## 4. Evaluation

To validate the efficacy of our cross-layer analysis framework (Section 3), we conducted an end-to-end security investigation of seven production automotive systems, including six IVI platforms from major manufacturers (Mercedes-Benz, Tesla, Denza, FAW-VW, SAIC, Bosch) and one ADAS module (Minieye). Our goal was to answer a concrete security question: Can an adversary chain hardware-level firmware access with web-layer vulnerabilities to achieve fleet-wide backend compromise?

Across these systems, we uncovered 23 previously unknown vulnerabilities, 7 of which received CVE identifiers following responsible disclosure. Most critically, we constructed a full attack chain against Mercedes-Benz’s IoV infrastructure that enables remote control of approximately 6 million vehicles via TCU command injection. The following subsections detail our experimental setup, vulnerability findings, and real-world impact—collectively substantiating the practical value of our methodology.

### 4.1. Experimental Setup

Our research was conducted in strict adherence to responsible disclosure protocols, ensuring that all findings were reported to and verified by the vendors. We evaluated 7 systems in total: 6 IVI platforms—Mercedes-Benz (Stuttgart, Germany), Tesla (Austin, TX, USA), Denza (Shenzhen, China), FAW-VW (Changchun, China), SAIC (Shanghai, China), Bosch (Gerlingen, Germany)—and one ADAS module—Minieye (Shenzhen, China). Four vendors permitted web-layer fuzzing (Mercedes-Benz, Denza, Tesla, FAW-VW); others restricted it due to DoS concerns. Where allowed, we limited request rates (100 requests/run, 0.5 s interval) to minimize service disruption.

Our symbolic execution used Angr [21] to generate initial seeds. Web fuzzing combined boofuzz [22] with a modified SNIPUZZ [20] that uses text similarity (via Euclidean distance on response embeddings) as a lightweight coverage proxy.

### 4.2. End-to-End Attack Chain: Compromising Mercedes-Benz’s IoV Backend

We demonstrate the practical impact of our framework through a complete attack chain against Mercedes-Benz’s connected vehicle ecosystem. The chain begins with physical access to the Head Unit (NTG-55), where we bypassed secure boot and storage encryption by reverse-engineering CAN-bus messages and extracting firmware via NAND flash disassembly of the HERMES Telematics Control Unit (TCU) (Qualcomm MDM9615). This yielded hard-coded credentials and regional TLS certificates used for backend authentication.

Leveraging an active e-SIM extracted from the TCU, we established a 4G connection to the carrier’s private APN network after spoofing the IMEI to evade ICCID–IMEI binding checks. With valid certificates and network access, we reached internal backend services. There, we exploited an SSRF vulnerability in a social media integration component to gain arbitrary file read and ultimately command execution on the central IoV server.

This access enabled us to invoke TCU APIs controlling critical vehicle functions—including door locks, ignition, headlights, and windows—across all Mercedes-Benz vehicles connected to the platform. We verified this capability on six in-house vehicles (with owner consent). At the time of testing, the compromised backend served approximately 2 million accounts in our region, consistent with public reports of  6 million global connected vehicles [23].

Additional vulnerabilities in the same ecosystem included unauthenticated user data exposure (names, registration times via phone number lookup) and Swagger API documentation leakage under /v3/api-docs, which accelerated endpoint discovery.

### 4.3. Cross-Vendor Vulnerability Landscape

Beyond Mercedes-Benz, our methodology revealed systemic weaknesses across the automotive supply chain. Table 1 summarizes key findings across all evaluated vendors. Common patterns include:

- Hardcoded credentials in frontend JavaScript (SAIC) or firmware (Denza);
- Unauthenticated or misconfigured backend services exposed to the public internet (FAW-VW, Minieye, Denza);
- Weak isolation between APN networks and internal services (Bosch);
- Broken access control enabling privilege escalation (Tesla’s employee portal via @tesla.com email registration).

Notably, multiple vendors allowed attackers with minimal physical access (e.g., USB or SD card) to extract secrets or bypass hardware encryption—highlighting the fragility of local trust assumptions in connected vehicles.

As shown in Table 1, while some vulnerabilities require physical access to extract initial secrets (e.g., Mercedes-Benz firmware decryption via SD card), the majority of high-impact flaws—including SSRF, broken API access controls, and public backend exposure—are fully remote. Notably, attackers need only a valid user account (obtainable via phishing or app compromise) to exploit these fleet-wide vulnerabilities. This demonstrates that our threat model’s inclusion of physical access is conservative: even under a purely remote attacker assumption, critical risks persist.

### 4.4. Real-World Impact and Responsible Disclosure

Our evaluation prioritizes actionable, high-impact findings—this approach yielded three concrete outcomes:

1. Seven vulnerabilities were assigned CVE identifiers (Table 2), including CVE-2019-19558 (CVSS 7.5)—an SSRF vulnerability in the Mercedes-Benz backend that enabled arbitrary file reads and unauthorized access to internal vehicle systems.
2. All affected vendors acknowledged the issues and deployed fixes within one month of disclosure.
3. We demonstrated a scalable attack path affecting millions of vehicles, underscoring the urgency of securing the IoV backend.

We recognize that traditional evaluation metrics (e.g., code coverage, bug count per hour) are poorly suited for closed-source, heterogeneous automotive systems. Instead, the value of our work lies in exposing exploitable, end-to-end attack paths that would remain hidden under modular or black-box testing. While standardized benchmarks may emerge in the future, our results show that a holistic, investigation-driven methodology is essential for uncovering real-world risks in the connected vehicles.

## 5. Discussion

In this section, we detail the experiences, challenges, and revelations encountered throughout our exploration of IVI system vulnerabilities, aiming to illuminate pathways for future research and development in enhancing IVI system security.

### 5.1. Limitations

While our research and framework have provided valuable insights into IVI vulnerabilities, it is crucial to acknowledge its limitations, as they delineate the scope and applicability of our findings. The symbolic execution engine, while robust in certain scenarios, was incapable of incorporating Android APKs, necessitating manual recovery of URLs and request bodies via reverse engineering. This manual intervention, albeit necessary, raises concerns about scalability and replicability in larger, more complex systems. Additionally, our black-box approach to web application fuzzing testing, while effective in certain respects, did not allow for precise coverage information to be garnered. Our web fuzzing was intentionally limited in request numbers to protect the production environment and uphold its online status. Furthermore, the verification of fuzzing results and the exploitation of the discovered vulnerabilities were non-trivial due to inherent challenges in both hardware and software domains, potentially limiting the depth and applicability of our findings. In addition, the investigation of IVI system vulnerabilities required an extensive manual effort, especially in firmware analysis and the examination of the communication protocol. The complexity and obfuscation inherent in code and proprietary communication protocols not only heightened the challenge but also raised pertinent questions about the transparency and accessibility of cybersecurity research within the automotive domain.

Moreover, our analytical framework is primarily designed for cloud-connected IVI systems with centralized backend architectures. It does not currently address vehicle-to-vehicle (V2V) or vehicle-to-infrastructure (V2I) communication channels, nor does it model threats arising from on-board AI perception or control stacks in autonomous driving scenarios. As such, the framework’s applicability is bounded to the attack surface of human-operated, internet-connected vehicles.

Future enhancements could extend the model by incorporating zero-trust principles across the vehicle-cloud boundary, integrating runtime integrity monitoring for IVI processes, or adapting the methodology to support edge-based AI deployments. However, such extensions would require access to additional interfaces and threat models beyond the scope of this work.

Furthermore, our evaluation was conducted primarily on vehicle systems and backend infrastructures deployed within a specific geographic region (e.g., the Mercedes-Benz backend serving approximately 2 million local accounts). Due to regional compliance requirements (such as GDPR in Europe or DSL in China), automotive vendors often fragment their backend architecture; thus, the specific vulnerabilities identified here may manifest differently in other regions. Finally, our firmware extraction methodology relies on hardware-specific techniques (e.g., NAND flash disassembly), which requires physical access to a representative unit and adaptation to distinct hardware board revisions, preventing fully automated application across all vehicle models.

### 5.2. Lessons Learned

Our investigation underscores a fundamental tension in the design of modern connected vehicles: the pursuit of seamless user experience and centralized fleet management has inadvertently created high-value, single-point attack surfaces that undermine both privacy and safety at scale. The most consequential lesson is that today’s IoV architectures often conflate connectivity with controllability—granting backend services sweeping authority over vehicle functions without sufficient cross-checks or defense-in-depth. As demonstrated by our end-to-end compromise of Mercedes-Benz’s infrastructure, access to a single internal API—enabled by a chain starting from physical firmware extraction—translated into remote control over millions of vehicles worldwide. This reveals a systemic architectural flaw: centralized cloud platforms, while operationally efficient, become crown jewels whose compromise invalidates local security measures across an entire fleet.

This risk is exacerbated by the mismatch between automotive lifecycles and software security practices. Vehicles remain on the road for 10–15 years, yet many rely on static credentials, unpatched libraries, or debug interfaces that were never designed for long-term exposure. The presence of Swagger documentation (/v3/api-docs) in production backends or hardcoded secrets in JavaScript bundles (e.g., SAIC) illustrates how development-time conveniences become persistent liabilities in deployed systems. Moreover, the supply chain complexity—spanning OEMs, Tier-1 suppliers like Bosch, and ADAS vendors like Minieye—further fragments accountability, allowing vulnerabilities to persist at integration boundaries.

Moreover, our work challenges the traditional boundary between “safety-critical” and “infotainment” domains. Once considered isolated, IVI systems now serve as authenticated gateways to cloud-managed actuators (doors, ignition, lights). An attacker need not breach the CAN bus directly; compromising the IVI-to-cloud trust chain is sufficient. This demands a paradigm shift: security can no longer be siloed within individual ECUs or layers. Instead, the IoV must be treated as an end-to-end, cross-layer ecosystem where trust is continuously verified—not assumed based on network location, device ownership, or physical access. Only through such a holistic rethinking can the industry reconcile the promise of connected mobility with its inherent security obligations.

While our study centers on connected vehicles operated by human drivers—a category representing the vast majority of today’s IoV deployments—the security failures we expose carry heightened societal implications as the industry transitions toward autonomous mobility services. Emerging research highlights that AVs are increasingly envisioned as mobility solutions for non-driving populations, including children, the elderly, and persons with disabilities [31,32,33]. In such contexts, remote compromise of backend infrastructure could place users at acute risk, as they may lack the capacity to detect anomalies or intervene during an attack. Moreover, AI-driven vehicle functions often depend on cloud-mediated perception and control pipelines [34,35,36], which inherit the same trust assumptions and vulnerabilities that we identified in current IVI–cloud architectures. Although a full security analysis of autonomous-vehicle-specific stacks, including sensor fusion and real-time decision-making, lies beyond the scope of this paper, our findings highlight the urgent need to harden the cloud-facing interfaces that will support future autonomous ecosystems.

### 5.3. Mitigations

Our analysis reveals that the root cause of fleet-wide compromise lies not in isolated implementation bugs, but in architectural assumptions that conflate physical access with trust and treat internal networks as inherently safe. To disrupt the attack chains we demonstrated—such as extracting hardcoded credentials from IVI firmware to exploit SSRF in cloud backends—vendors must adopt a defense-in-depth strategy grounded in zero-trust principles across the vehicle-to-cloud stack.

At the firmware level, sensitive artifacts such as APN configurations, TLS certificates, and backend authentication tokens must never be stored in plaintext within recoverable filesystem images. Instead, these secrets should be bound to hardware-backed secure elements (e.g., Trusted Execution Environments or dedicated HSMs), ensuring they cannot be extracted even if an attacker gains physical access to storage chips. Secure boot mechanisms, rigorously verified from bootloader to application layer, are essential to prevent unauthorized firmware repackaging—a vector we exploited in multiple vendors. Furthermore, all over-the-air (OTA) updates must be cryptographically signed and validated prior to installation, a practice that remains inconsistently applied despite its critical role in supply chain integrity [23].

On the network side, the assumption that carrier-provisioned APNs provide sufficient isolation is demonstrably flawed. As shown in our Mercedes-Benz case study, attackers can spoof IMEI/ICCID pairs to gain access to private APN networks and reach internal services. Vendors should collaborate with mobile carriers to enforce strict APN whitelisting and device binding policies. Additionally, outbound traffic from vehicles should be restricted via per-device firewall rules that permit communication only with a minimal set of authenticated endpoints, thereby limiting lateral movement even if one vehicle is compromised.

For backend services, the most urgent priority is to eliminate implicit trust in vehicle-originated requests. Internal APIs—even those behind authentication gateways—must assume every call could be malicious. This includes deploying mutual TLS (mTLS) with vehicle-specific client certificates for all TCU-facing endpoints, rather than relying on session tokens or IP-based allowlists. SSRF protections must explicitly block resolution to private IP ranges (e.g., 10.0.0.0/8) and internal service names, a gap that enabled full server takeover in our evaluation. Debug interfaces such as Swagger UI (/v3/api-docs) should be disabled in production, as their exposure significantly accelerates attacker reconnaissance. Finally, access control policies must be fine-grained: commands affecting critical functions like door locks or engine start should require explicit ownership verification and contextual risk signals (e.g., geolocation consistency).

Looking ahead, emerging techniques may further strengthen these defenses. For instance, large language models show promise in automating vulnerability detection during development or enhancing code review processes [37,38,39], while machine learning–based anomaly detection could flag abnormal backend request patterns in real time [40]. However, no amount of automation substitutes for sound architecture. The vulnerabilities we uncovered are fundamentally design-level failures—and their mitigation requires rethinking trust boundaries in the Internet of Vehicles.

## 6. Conclusions

In conclusion, our study reveals significant security vulnerabilities in in-vehicle infotainment systems and web services within the IoV ecosystem. These vulnerabilities, when exploited, can lead to widespread control over connected vehicles, compromising safety and security. We identified novel vulnerabilities affecting 7 major manufacturers, potentially impacting seven million consumers globally with our evaluation framework. All vulnerabilities have been responsibly disclosed and assigned CVEs.

## Figures and Tables

**Table 1:** Summary of Identified Vulnerabilities, Their Impacts, and Proposed Mitigation Across Automobile Manufacturers.

Manufacturer	Attack Vector	Vulnerability	Impact	Mitigations
Mercedes-Benz	Physical access (SD card, e-SIM)	Bypassing hardware encryption Server side SSRF Brute forcing credentials for SSH	Get firmware Arbitrary server-side file read Get shell to control all cars	Use HSM for secrets Prevent SSRF Validate end-user requests
	Remote (API leakage)	Broken access control	Phone number → user info leak (name, registration time)	Restrict API access, add authentication
Denza	Physical (USB), Remote (web)	Directory traversal → shadow file Broken Redis access control	Get shell Car location exposure Push firmware upgrade	Verify firmware signatures Fix directory traversal Secure Redis
Bosch	Physical (e-SIM)	Breaking APN network isolation	Expose internal services	Remove irrelevant services
Minieye ADAS	Remote (web)	Broken access control	ML training images publicly accessible	Move to intranet with auth
Tesla	Remote (web)	Broken access control	@tesla.com email registration → employee services access	Block @tesla.* registration Email verification
FAW-VW	Remote (web, auth)	Stored XSS in ad title	JS execution for backend admins → info leak	Filter XSS characters
		Public backend service Weak passwords	Control 200 cars (horn, engine, locks)	Strong passwords, intranet only
SAIC	Remote (web, auth)	Public backend via APK analysis Hardcoded JS password = SSH password	End-user information leak	Backend passwords, no reuse

* Attack vectors: Physical = local interface access (e.g., USB/OBD-II); Remote = internet-reachable with user token.

**Table 2:** Identified Threats in Mercedes-Benz’s IoV System.

CVE ID	Vulnerability Type	Score	Severity
CVE-2019-19556 [24]	Authentication Bypass	4.6	Medium
CVE-2019-19560 [25]	Authentication Bypass	4.6	Medium
CVE-2019-19562 [26]	Authentication Bypass	4.6	Medium
CVE-2019-19557 [27]	Misconfiguration	2.4	Low
CVE-2019-19561 [28]	Misconfiguration	2.4	Low
CVE-2019-19563 [29]	Misconfiguration	2.4	Low
CVE-2019-19558 [30]	SSRF	7.5	High

**Figure A1:**
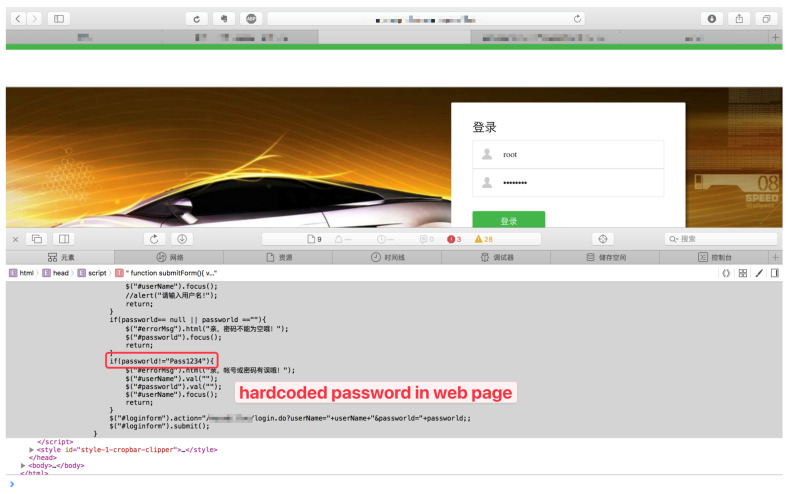
Exposure of Backend Services through Hardcoded Credentials in SAIC’s Frontend Javascript.

**Figure A2:**
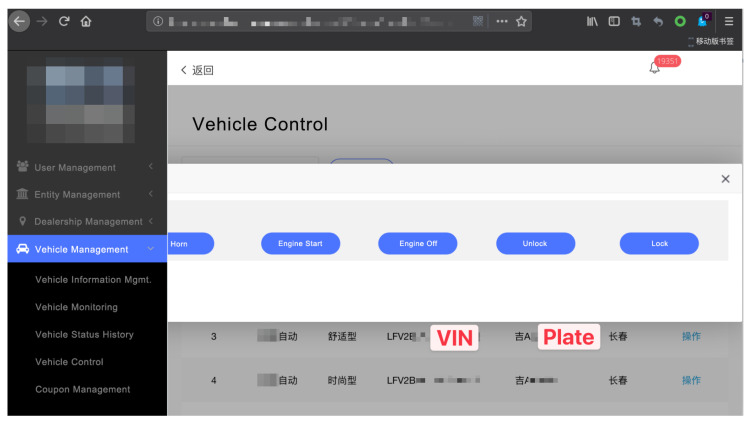
Potential for Unauthorized Vehicle Control via Brute Force Attack on FAW VW’s Backend Services.

**Figure A3:**
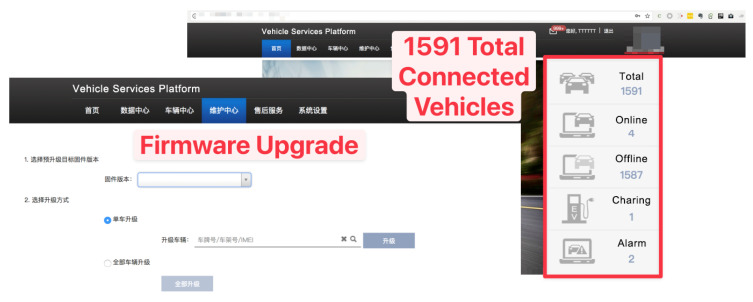
Risk of Unauthorized Vehicle Access and Firmware Manipulation due to Denza’s Weak Password Protocol.

**Figure A4:**
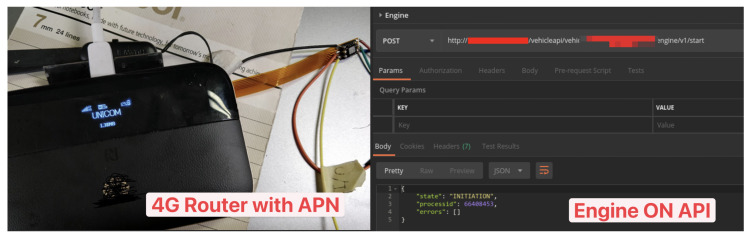
Remotely Start Mercedes-Benz Vehicle Engine Using Exposed Backend API with APN Access.

## Data Availability

The original contributions presented in this study are included in the article. Further inquiries can be directed to the corresponding author.

## Abbreviations

The following abbreviations are used in this manuscript:

IVI	In-Vehicle Infotainment
IoV	Internet of Vehicles
TCU	Telematics Control Unit
TSP	Telematics Service Provider
APN	Access Point Name
SSRF	Server-Side Request Forgery
OTA	Over-The-Air
TEE	Trusted Execution Environment
HSM	Hardware Security Module
mTLS	mutual Transport Layer Security
CAN	Controller Area Network
V2X	Vehicle-to-Everything

## Appendix A

The exploration of various vulnerabilities in connected vehicle backend services, as detailed in the appendix, underscores the criticality of robust cybersecurity practices in vehicular technologies. The SAIC vulnerability (Figure A1) exposes the perils of hardcoded credentials, enabling unauthorized access and potential data manipulation. Similarly, the FAW VW vulnerability (Figure A2) demonstrates the tangible threats posed by weak credentials, where attackers can remotely control vehicle functionalities, posing a direct threat to user safety. The Mercedes-Benz SSRF vulnerability allows us to view the source code of the backend server and even remotely control up to 6 million connected vehicle as shown in Figure A4. Lastly, the Denza vulnerability (Figure A3) highlights the catastrophic potentials when backend services, controlling multiple vehicles, are compromised due to weak password protocols, enabling unauthorized firmware updates and vehicle control. These vulnerabilities collectively emphasize the the impact of the vulnerabilities that we have found using our framework.

### Appendix A.1. Mercedes-Benz SSRF Vulnerability

The aforementioned Mercedes-Benz exploitation chain allows us to perform SSRF attack with our customized 4G router and modified APN configuration. We further found internal APIs by reviewing the code base that is stored on the backend server. Utilizing both, we can remotely start Mercedes-Benz vehicle by just invoking the corresponding APIs, as shown in Figure A4.

### Appendix A.2. SAIC Access Control Vulnerability

Hardcoded credentials in the frontend Javascript of SAIC backend services present a glaring security loophole, as illustrated in Figure A1. Attackers exploiting this vulnerability gain unauthorized access to backend services, potentially manipulating, stealing, or corrupting data. Moreover, the exposure of sensitive user data and system controls can lead to widespread malicious activities, such as unauthorized transactions or triggering of unintended actions in connected systems.

### Appendix A.3. FAW VW Weak Password Vulnerability

The weak credential of FAW VW backend services, depicted in Figure A2, is not merely a lapse in secure coding practices but a direct enabler for attackers to manipulate connected vehicles. By brute-forcing the credentials, attackers can query and dispatch commands to vehicles remotely, using VIN or plate numbers as identifiers. The potential for malevolent actors to lock and unlock doors, or even control engine states, poses not only a privacy concern but a tangible threat to physical safety and security of the vehicle users.

### Appendix A.4. Denza Access Control Vulnerability

Denza auto, a notable automobile manufacturer in China, manifested a discernible vulnerability on its USB interface. Specifically, the interface was capable of autonomously virtualizing the Modem device and achieving recognition from the connected PC, circumventing the necessity for third-party driver installation. This vulnerability facilitates unauthorized access to the interface via AT command operating software, thereby revealing pivotal login parameters, such as the Access Point Name (APN), and potentially exposing the system to malicious interventions.

Interestingly, an unauthenticated Redis service was also identified to be accessible devoid of any requisite authentication protocols. Notably, prior unauthorized access was evident indicating a potential security breach and underscoring the importance of enforcing stringent authentication mechanisms for pivotal services to mitigate security risks.

The Denza vulnerability, affecting up to 1591 connected vehicles and illustrated in Figure A3, is not merely a risk but a potential catastrophe waiting to happen. The backend service, susceptible to unauthorized access due to weak password protocols, can be exploited to control connected vehicles and even perform firmware upgrades. Attackers can manipulate vehicle functionalities, disable safety features, or introduce malicious firmware updates, thereby jeopardizing the safety and operability of the vehicles and potentially causing large-scale incidents.

### Appendix A.5. GMW GROUP Insufficient Access Control and Broken Authentication Vulnerability

GMW GROUP, a recognized automotive brand in China, demonstrated a notable vulnerability concerning Access Point Name (APN) network access. Typically, when a mobile phone’s APN is inaccurately configured, the network operator will nonetheless accept the user’s network connection request, utilizing the default APN for network login. Although BOSCH’s TBOX did not retrieve the specific APN name in this instance, the operator’s connection to the default network was indeed connected to the private network APN. This implies that, following the attacker arbitrarily setting an APN, the default network connected is the intranet, necessitating the provision of an accurate public network APN for public network connection. A significant misstep was observed with China Unicom operators in their selection of access to the APN network.

Upon scanning the intranet, a plethora of servers were revealed, with numerous servers having open ports 22, 80, 443, and so forth. Our IP scan was solely for subnet segment 60, with ports 22–500. Notably, the Great Wall does not enumerate the access IPs permitted by TBOX, and the absence of a whitelist results in TBOX accessing a substantial number of servers unrelated to its business, thereby introducing potential security risks and underscoring the necessity for meticulous IP whitelisting and port management to safeguard against unauthorized access and potential malicious activities.
